# An Unusual Cause of Refractory Bleeding in Cirrhosis

**DOI:** 10.14309/crj.0000000000001498

**Published:** 2024-09-12

**Authors:** Meagan Alvarado, Darrick K. Li

**Affiliations:** 1 Department of Medicine, Section of Digestive Diseases, Yale School of Medicine, New Haven, CT

**Keywords:** cirrhosis, acquired inhibitor, gastrointestinal bleeding

## Abstract

Acquired hemophilia A (AHA) is a rare bleeding disorder caused by the development of antibodies against factor VIII. AHA has previously been reported in association with malignancy and autoimmune disorders, but rarely with liver disease. A prolonged activated partial thromboplastin time is the initial laboratory manifestation of this condition but may be challenging to interpret in the setting of abnormal markers of coagulation typically seen in cirrhosis. We present a case of AHA in a patient with decompensated cirrhosis resulting in refractory bleeding and highlight the complexities of interpreting abnormal coagulation factors in patients with cirrhosis.

## INTRODUCTION

Acquired hemophilia A (AHA) is characterized by the spontaneous development of neutralizing autoantibodies against the endogenously produced coagulation factor, factor VIII.^1,2^ It is a rare disorder with an incidence in the general population of approximately 1.5 cases per million person years and has previously been described in association with a variety of conditions including malignancy, infections, and autoimmune disorders including systemic lupus erythematosus and rheumatoid arthritis. AHA has very seldomly been reported in patients with liver disease apart from several cases in patients with chronic hepatitis C virus infection.^3–6^ An isolated prolonged activated partial thromboplastin time (PTT) is often the first laboratory finding in conjunction with AHA. However, this can be challenging to detect in patients with underlying abnormal coagulation tests in the setting of the coagulopathy of chronic liver disease. We present a case of AHA in a patient with decompensated cirrhosis that highlights these challenges.

## CASE REPORT

**Figure 1.**
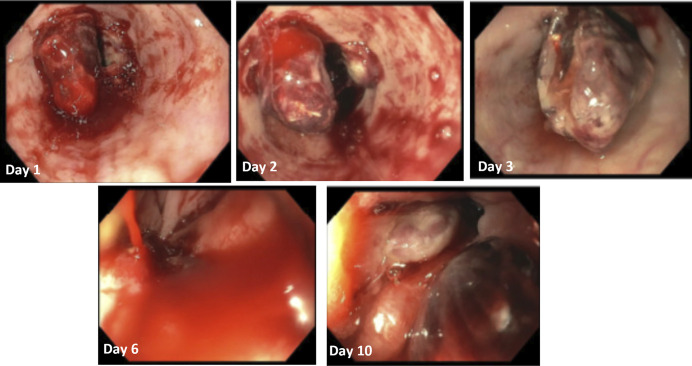
Endoscopic findings during hospitalization.

A 60-year-old woman with alcoholic cirrhosis, decompensated by ascites and portosystemic encephalopathy, hepatitis C negative, prior medical history of hypertension, diverticulosis, Barrett's esophagus, and gastric antral vascular ectasias, presented to our hospital with hematemesis and hematochezia. One week before presentation, the patient underwent an upper endoscopy for evaluation of symptomatic anemia and was found to have large esophageal varices and underwent endoscopic variceal ligation for primary variceal bleeding prophylaxis. On hospital admission, she was found to have a hemoglobin of 5.9 g/dL. Prothrombin time (PT) was 14.6 seconds, international normalized ratio was 1.38, and PTT was 87.8 seconds. The patient was not on anticoagulation or antiplatelet medications as an outpatient or throughout hospitalization. An upper endoscopy was performed on hospital day 1 and was notable for esophageal postligation ulcers with active oozing and was treated with hemostatic spray (Figure 1). On HD2, she had recurrent hematemesis and underwent repeat upper endoscopy on HD3 during which there was fresh blood in the esophagus with active bleeding in the distal esophagus near an esophageal ulcer treated with a hemostatic clip and hemostatic powder (Figure 1).

While the working diagnosis remained refractory variceal bleeding and transjugular intrahepatic portosystemic shunt placement was being considered, a further review of her laboratory studies before admission led to the observation that her PTT had been rising (independently of the PT) in the past several months before presentation (Table 1). Individual coagulation factor levels were assessed and were consistent with globally depressed factor levels secondary to chronic liver disease and vitamin K deficiency but with significantly depressed factor VIII activity. A mixing study did not show correction of the PTT suggesting the presence of an inhibitor, which was later confirmed with high titer of a factor VIII inhibitor, consistent with AHA.

**Table 1.** Relevant laboratory values

	Reference range	5 months before admission	1 week before admission	Day of admission	Day 6 of admission	Day 10 of admission
Complete blood count						
Hemoglobin (g/dL)	11.7–15.5	7.6	4.6	5.9	8.7	
Hematocrit (%)	35–45	24.0	15.4	18.1	24.7	
White blood cell (10^9^/L)	4.0–11.0	8.4	8.5	14.4	15.8	
Platelets (10^9^/L)	150–450	162	308	172	115	
Coagulation studies						
PT (s)	9–14	13.2	12.3	14.6	13.0	11.2
PTT (s)	22–35	36.3	74.6	87.8	72.5	>139.0
INR		1.31	1.23	1.38	1.22	1.03
Individual factor levels						
Factor II activity	56%–108%				44.5	
Factor V activity	65%–136%				55.4	
Factor VIII activity	66%–143%				3.3	
Factor IX activity	70%–142%				57.3	
Factor XI activity	67%–127%				30.4	
Factor XII activity	73%–145%				36.9	
Factor VIII chromogenic	50%–150%				<11.2	
Mixing study						
PT 1:1 Mix 0”	9–14				11.9	
PT 1:1 Mix control 0”					12.2	
PTT 1:1 Mix 0”	22–35				47.8	
PTT 1:1 Mix 60”					61.0	
PTT 1:1 Mix control 0”	22–35				27.3	
PTT 1:1 Mix control 60”					29.6	
Inhibitor assay						
Factor VIII inhibitor (Bethesda units)	0				34	

INR, international normalized ratio; PT, prothrombin time; PTT, partial thromboplastin time.

On HD6, the patient was started on factor eight inhibitor bypassing activity and daily intravenous vitamin K with clinical improvement in bleeding. However, she developed persistent bleeding on HD10 and was started on solumedrol, recombinant factor VIIa, and tranexamic acid infusion with plans for further immunosuppression with cyclophosphamide, rituximab, or emicizumab. Despite these interventions, she continued to have recurrent bleeding from esophageal ulceration, required in total 10 units of packed red blood cells, 20 units of cryoprecipitate, 3 units of plasma, and underwent a total of 4 upper endoscopies during which her esophageal bleeding was managed with sclerotherapy with ethanolamine, application of hemostatic clips, and additional applications of hemostatic spray. Empiric left gastric artery embolization was performed with interventional radiology without clinical improvement. She ultimately developed diffuse spontaneous bleeding from multiple sites, and she was eventually transitioned to comfort measures and died on HD17.

## DISCUSSION

To our knowledge, this is the first case report of AHA in a patient with decompensated cirrhosis. This case highlights not only a rare cause of refractory bleeding in patients with chronic liver disease as well as the complexities of interpreting abnormal coagulation factors in patients with cirrhosis.

Isolated prolonged PTT is the most observed lab abnormality seen in patients with AHA, although lupus anticoagulant and heparin exposure/contamination can result in an isolated prolonged PTT. If suspicion is high, a mixing study is needed to differentiate between deficiency, which will correct with addition of normal control plasma, vs an inhibitor, which will not correct. Treatment of active bleeding in AHA includes infusion of large doses of factor VIII concentrates or administration of diamino-8-D-arginine vasopressin (ddAVP).^7^ In patient with high-titer inhibitors, activated prothrombin complex concentrate or recombinant factor VIIa is used to bypass factor VIII effects in the coagulation cascades, and plasmapheresis can be considered.^2,7^ Inhibitor eradication therapy with glucocorticoids and adjuvant agents including cyclophosphamide, azathioprine, vincristine, mycophenolate, and 2-chlorodeoxyadenosine have all been used with up to 60%–70% response rate.^1^ However, response with these agents can take weeks to months; therefore, they are not indicated as the primary approach to acute bleeding.

In our case, although the acquired factor VIII inhibitor was identified at the time of refractory bleeding, her PTT had been gradually uptrending for weeks leading up to the event. These abnormal lab trends went unrecognized as the patient's abnormal coagulation studies were presumed to be secondary to her underlying cirrhosis. It is important to note that PT and PTT are generally elevated to a similar degree in cirrhosis. Interestingly, factor VIII levels tend to be preserved until late in chronic liver disease as nitric oxide and prostacyclin released in the setting of liver disease results in elevated factor VIII levels.^8^ This accounts for the often normal or near normal PTT in early liver disease, despite elevations in PT and international normalized ratio.

Although acquired factor inhibitors are rare in the general population, it is likely that such cases have been overlooked in patients with chronic liver disease as abnormal coagulation parameters are often attributed to the coagulopathy of cirrhosis. In addition, critically ill patients with cirrhosis are prone to developing disseminated intravascular coagulopathy, which can also present with refractory bleeding and mimic the presentation of acquired hemophilia. It is imperative that the clinicians consider the broad range of coagulopathic processes in patients with cirrhosis, as early identification and directed treatment may drastically change clinical outcomes.

## DISCLOSURES

Author contributions: M. Alvarado and DK Li conceived of the project and were involved in the clinical care of the patient; M. Alvarado drafted the manuscript, and both authors were involved with editing and actualization of the manuscript to its final form. DK Li is the article guarantor.

Financial disclosure: None to report.

Informed consent was obtained for this case report.
